# Supplementary material for: Changes Over a 10‐Year Period in the Distribution Ranges and Genetic Hybridization of Three Pelophylax Pond Frogs in Central Japan
Source: Ecol Evol. 2025 Aug 7;15(8):e71856. doi: 10.1002/ece3.71856 (PMC12329352; doi:10.1002/ece3.71856)
Supplement: Supplementary file 2 — Figure S1. Environmental analyses for the two basins over the last few decades, that is, temperature and precipitation. Fifty years of observational data of temporal variations in temperature and precipitation from 1970 to 2020 in the Matsumoto (Matsumoto Meteorological Observatory) and Ina Basins (Iida Meteorological Observatory). Analyses of temporal variations in temperature and precipitation in the Matsumoto Basin and Ina Basin from 1970 to 2020 using the STL decomposition method. The data, trend components, seasonal components, and residual components are shown for (a) the daily maximum temperature in the Matsumoto Basin, (b) the daily maximum temperature in the Ina Basin, (c) the daily minimum temperature in the Matsumoto Basin, (d) the daily minimum temperature in the Ina Basin, (e) the daily mean temperature in the Matsumoto Basin, (f) the daily mean temperature in the Ina Basin, (g) the monthly precipitation in the Matsumoto Basin, and (h) the monthly precipitation in the Ina Basin. The top row of each figure shows the original data and trend components, the middle row shows the seasonal components, and the bottom row shows the reminder components. The box highlights in the figures indicate the survey years, with 1980, 2010, and 2020 highlighted, respectively. It became clear that due to the effects of global warming and/or urbanization (described below), the trends in the monthly average daily maximum temperatures, monthly average daily minimum temperatures, and monthly average daily temperatures in both basins have all been gradually rising over the past 50 years. Precipitation varies greatly within each year, with no clear trends observed over the past 50 years. [file ECE3-15-e71856-s002.pdf]

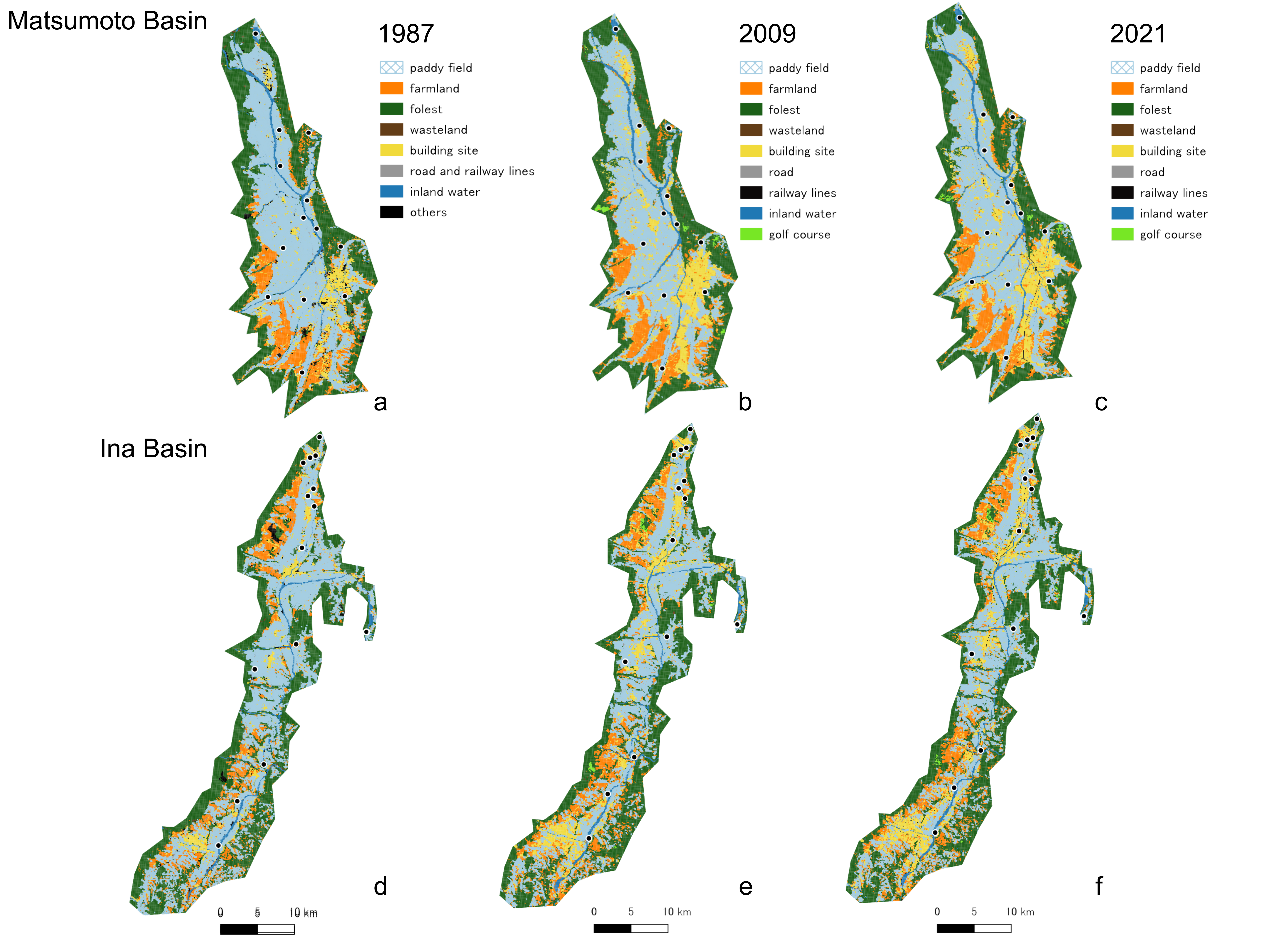

Figure S1

Environmental analyses for the two basins over the last few decades, i.e., temperature and precipitation. Fifty years of observational data of temporal variations in temperature and precipitation from 1970 to 2020 in the Matsumoto (Matsumoto Meteorological Observatory) and Ina Basins (Iida Meteorological Observatory). These analyses of temporal variations using the STL decomposition method. Analyses of temporal variations in temperature and precipitation in the Matsumoto Basin and Ina Basin from 1970 to 2020 using the STL decomposition method. The data, trend components, seasonal components, and residual components are shown for (a) the daily maximum temperature in the Matsumoto Basin, (b) the daily maximum temperature in the Ina Basin, (c) the daily minimum temperature in the Matsumoto Basin, (d) the daily minimum temperature in the Ina Basin, (e) the daily mean temperature in the Matsumoto Basin, (f) the daily mean temperature in the Ina Basin, (g) the monthly precipitation in the Matsumoto Basin, and (h) the monthly precipitation in the Ina Basin. The top row of each figure shows the original data and trend components, the middle row shows the seasonal components, and the bottom row shows the reminder components. The box highlights in the figures indicate the survey years, with 1980, 2010, and 2020 highlighted, respectively.

It became clear that due to the effects of global warming and/or urbanization (described below), the trends in the monthly average daily maximum temperatures, monthly average daily minimum temperatures, and monthly average daily temperatures in both basins have all been gradually rising over the past 50 years. Precipitation varies greatly within each year, with no clear trends observed over the past 50 years.
