# Supplementary material for: Changes Over a 10‐Year Period in the Distribution Ranges and Genetic Hybridization of Three Pelophylax Pond Frogs in Central Japan
Source: Ecol Evol. 2025 Aug 7;15(8):e71856. doi: 10.1002/ece3.71856 (PMC12329352; doi:10.1002/ece3.71856)
Supplement: Supplementary file 3 — Figure S2. Environmental analyses for the two basins over the last few decades, that is, land use changes from 1987 to 2021 in the both basins. (a) the Matsumoto Basin in 1987, (b) the Matsumoto Basin in 2009, (c) the Matsumoto Basin in 2021, (d) the Ina Basin in 1987, (e) the Ina Basin in 2009, (f) the Ina Basin in 2021. The results are shown color‐coded by land use type for each basin and each year. Land use types are assigned according to the priority of features in each 100 m mesh. Regarding land use, it was revealed that the area of paddy fields had decreased significantly over the 22 years from 1987 to 2009 (a 15.1% decrease in the Matsumoto Basin and a 17.4% decrease in the Ina Basin). In addition, although the decrease over the 12 years from 2009 to 2021 was less (a decrease of 5.2% in the Matsumoto Basin and 8.5% in the Ina Basin), which when averaged gives an annual rate of decrease that has been almost constant. Meanwhile, as the area of farmland and building areas has increased, it is believed that over the past 35 years there has been a great deal of land conversion from paddy fields to farmland and residential land, and from paddy fields to farmland and then residential land. [file ECE3-15-e71856-s003.pdf]

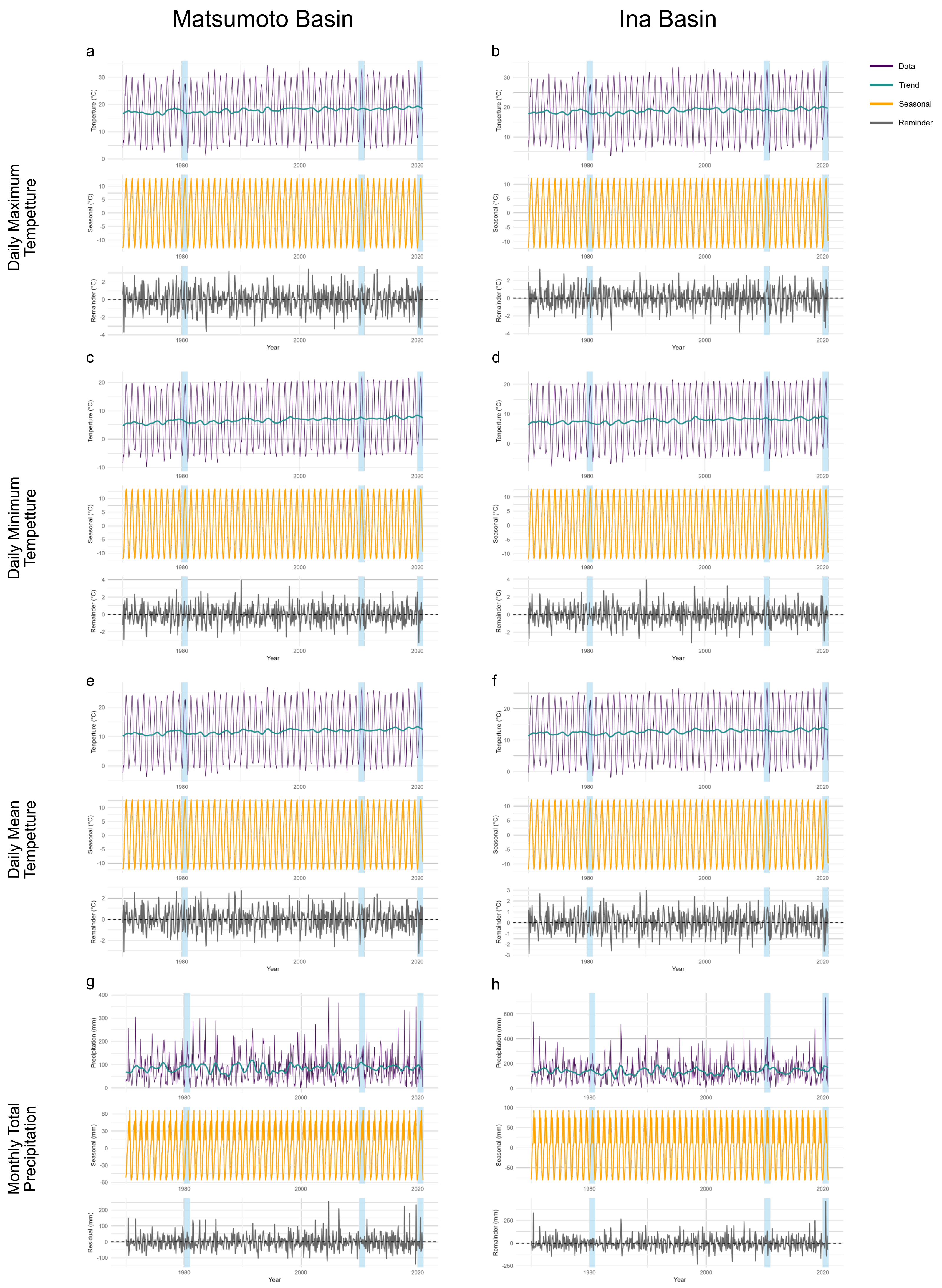

Figure S2

Environmental analyses for the two basins over the last few decades, i.e., land use changes from 1987 to 2021 in the both basins. (a) the Matsumoto Basin in 1987, (b) the Matsumoto Basin in 2009, (c) the Matsumoto Basin in 2021, (d) the Ina Basin in 1987, (e) the Ina Basin in 2009, (f) the Ina Basin in 2021.

The results are shown color-coded by land use type for each basin and each year. Land use types are assigned according to the priority of features in each 100 m mesh. Regarding land use, it was revealed that the area of paddy fields had decreased significantly over the 22 years from 1987 to 2009 (a 15.1% decrease in the Matsumoto Basin and a 17.4% decrease in the Ina Basin). In addition, although the decrease over the 12 years from 2009 to 2021 was less (a decrease of 5.2% in the Matsumoto Basin and 8.5% in the Ina Basin), which when averaged gives an annual rate of decrease that has been almost constant. Meanwhile, as the area of farmland and building areas has increased, it is believed that over the past 35 years there has been a great deal of land conversion from paddy fields to farmland and residential land, and from paddy fields to farmland and then residential land.
